# The Role of Biotics as a Therapeutic Strategy for Oral Mucositis - A Systematic Review

**DOI:** 10.1007/s12602-023-10116-z

**Published:** 2023-06-30

**Authors:** Leonor Frey-Furtado, Inês Magalhães, Maria João Azevedo, Benedita Sampaio-Maia

**Affiliations:** 1 Faculdade de Medicina Dentária, Universidade do Porto, Porto, Portugal; 2 i3S – Instituto de Investigação e Inovação em Saúde, Universidade do Porto, Porto, Portugal; 3 INEB – Instituto de Engenharia Biomédica, Universidade do Porto, Porto, Portugal; 4 Escola Superior de Biotecnologia – Universidade Católica Portuguesa, Porto, Portugal; 5 Academic Center for Dentistry Amsterdam, Vrije Universiteit Amsterdam and University of Amsterdam, Amsterdam, The Netherlands

**Keywords:** Oral mucositis, probiotics, prebiotics, oral cancer, head and neck cancer

## Abstract

**Objectives:**

Oral mucositis (OM) is an acute and highly prevalent side effect of cancer treatments. Currently, there is no effective strategy for its prevention or treatment. This systematic review aimed to assess the effectiveness of biotics used as a therapeutic strategy for the management of OM.

**Materials and Methods:**

The PRISMA checklist was followed and PubMed, Web of Science, and Scopus were screened for clinical and pre-clinical studies assessing the potential effects of biotics in OM. Inclusion criteria included in vivo studies related to oral mucositis evaluating the effect of biotics, and written in Portuguese, English, French, Spanish, or Dutch. The following exclusion criteria were used: systematic reviews and meta-analyses, reviews, case reports, opinion papers or comments, conference papers, letters without results, articles not related to oral therapy-induced mucositis or biotics, or in vitro articles that do not simulate oral mucositis.

**Results:**

From a total of 1250 articles retrieved, 9 were included in this systematic review. Four clinical studies reported a reduction in oral mucositis occurrence with *Lactobacillus* species (*Lactobacillus casei* and *Lactobacillus brevis* CD2) and *Bacillus clausii* UBBC07. In pre-clinical studies, *Lactococcus lactis* genetically modified and *Lactobacillus reuteri* reduced the severity of OM and *Streptococcus salivarius* K12 also decreased the size of the ulcers.

**Conclusion:**

The findings of this systematic review suggest that probiotic supplementation may potentially reduce the incidence of therapy-induced OM and decrease its severity in patients undergoing cancer treatment. However, the available evidence is marred by significant heterogeneity across studies.

## Introduction

Oral mucositis (OM) is an acute and highly prevalent side effect of cancer treatments, consisting of inflamed, erosive, or ulcerative lesions on the oral mucosa [1]. It is the result of a complex and dynamic combination of biological events, involving multiple pathways and interactions between cancer therapy and oral tissues [2]. According to a five-step pathogenesis model suggested by Sonis [3], radiation and chemotherapeutic drugs encourage tissue inflammation and cell apoptosis by producing harmful reactive oxygen species (Step 1 - Initiation) and activating transcription factors such as nuclear factor-B (Step 2 - Primary response). As a result, this will trigger a series of inflammatory pathways and cause proinflammatory cytokines to be upregulated (Step 3 - Amplification), culminating in ulceration (Step 4 - Ulceration). This step resolves when the extracellular matrix sends signals to the epithelium that impact cell proliferation and differentiation (Step 5 - Healing). The submucosa is then re-established, but not exactly to its prior state because of the mucotoxic injury inflicted by cancer therapy.

Although the incidence and severity of oral mucositis widely vary among patients and treatments prescribed, the mean incidence was reported to be approximately 80%, with several patients suffering from severe oral mucositis [4]. Patients who develop oral mucositis experience severe pain which interferes with their nutrition, quality of life (QOL), and ultimately, compliance with their treatment plan [5]. It has also been reported that patients with OM have twice the risk of developing infections and four times the risk of death compared to patients without OM [6]. The degree and duration of oral mucositis are related to the type of chemotherapy or radiation dose used, the volume of tissue treated, and the treatment duration [6].

Changes in the oral microbiome are also known to influence the incidence and severity of OM. This state of altered bacterial colonization associated with disease expression it is known as oral dysbiosis. Dysbiosis can be caused by genetic and environmental factors such as antibiotic use, diet alterations, stress, and chronic diseases [7]. The dominance of opportunistic microorganisms, such as *Candida* spp. and gram-negative bacteria, increases during cancer therapy and may further aggravate the inflammatory response [8].

According to the *Multinational Association of Supportive Care in Cancer and International Society of Oral Oncology Clinical Practice Guidelines for Oral Mucositis* [9], it is possible to mitigate the risk of developing OM by proceeding with prophylactic oral care, cryotherapy, anti-inflammatory agents (e.g. benzydamine mouthwash), photobiomodulation therapy (e.g. low-level laser therapy), and antimicrobial and coating agents [9]. In terms of the usual clinical interventions to minimize the impact of OM, these include basic oral care, the use of photobiomodulation, anesthetics (e.g. 2% viscous lidocaine mouth rinse), diet modification, and systemic opiates [6].

Despite these guidelines, the management of oral mucositis remains mostly symptomatic and there is no effective strategy for its prevention or treatment [10]. As so, the manipulation of the oral microbiome with biotics – probiotics, prebiotics, postbiotics, and symbiotics - emerged as an alternative treatment or co-adjuvant option. According to the World Health Organization (WHO), probiotics are defined as live microorganisms that confer a health benefit for the host when administered in adequate amounts. Besides probiotics, prebiotics are dietary molecules that promote the growth of beneficial bacteria, postbiotics are microbial metabolites that have beneficial effects, and symbiotics are a combination of pre-, pro-, or postbiotics [11]. Recently, there has been an increasing interest in their use to prevent, mitigate, or treat specific diseases, such as acute infectious diarrhea in infants [12] and periodontal disease [13].

Regardless of the positive effects of biotics in other diseases, the effect of the use of biotics on the management of therapy-induced oral mucositis in cancer patients is yet to be unveiled. Given this scenario, this paper aims to systematically revise the effectiveness of biotics as an alternative therapeutic strategy for the management of oral mucositis.

## Materials and Methods

### Protocol and Registration

This review was conducted following the Preferred Reporting Items for Systematic Reviews and Meta-Analyses (PRISMA) checklist and registered on the PROSPERO website, CRD42022314339.

### Information Sources and Search Strategy

To fulfill the goal of this systematic review, a PICO (population, intervention, comparison, and outcomes) question was formulated: What is the effect of biotics, compared to not using biotics, on the management of therapy-induced oral mucositis in cancer patients?

To develop this review, three databases were used: Pubmed, Scopus, and Web of Science, using the following search query: “(mucositis[MeSH Terms] OR oral mucosit* OR oromucosit*) AND (probiotics[MeSH Terms] OR prebiotics[MeSH Terms] OR probiotic* OR pro-biotic* OR prebiotic* OR pre-biotic* OR postbiotic* OR post-biotic* OR symbiotic* OR Lactobacillus OR Bifidobacterium OR Streptococcus OR Enterococcus OR Saccharomyces OR Lactococcus)”. Searches were conducted on December 14th, 2022.

### Eligibility Criteria

Inclusion criteria included studies related to oral mucositis, in vivo studies (in humans and animals), evaluating the effect of pre-, pro-, post-, and symbiotics, and written in Portuguese, English, French, Spanish, or Dutch.

The exclusion criteria were the following: systematic reviews and meta-analyses, reviews, case reports, opinion papers or comments, conference papers, letters without results, articles not related to oral therapy-induced mucositis, unrelated to pre-, pro-, post- or symbiotics, or in vitro articles that do not simulate oral mucositis.

### Selection Process

After removing duplicates, the titles and abstracts of the retrieved publications were independently reviewed by two reviewers (LF and IM). Studies not excluded in the screening phase were fully read and full-text analysis was independently conducted by the same reviewers. Any divergence was solved in discussion with a third-party (MJA and BSM). A total of 1250 articles were retrieved from bibliographic databases (PubMed, Scopus, and Web of Science). The study selection process is described in Fig. 2.

**Fig. 1:**
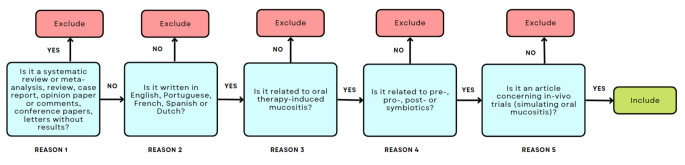
Workflow for Inclusion and Exclusion of Studies

**Fig. 2:**
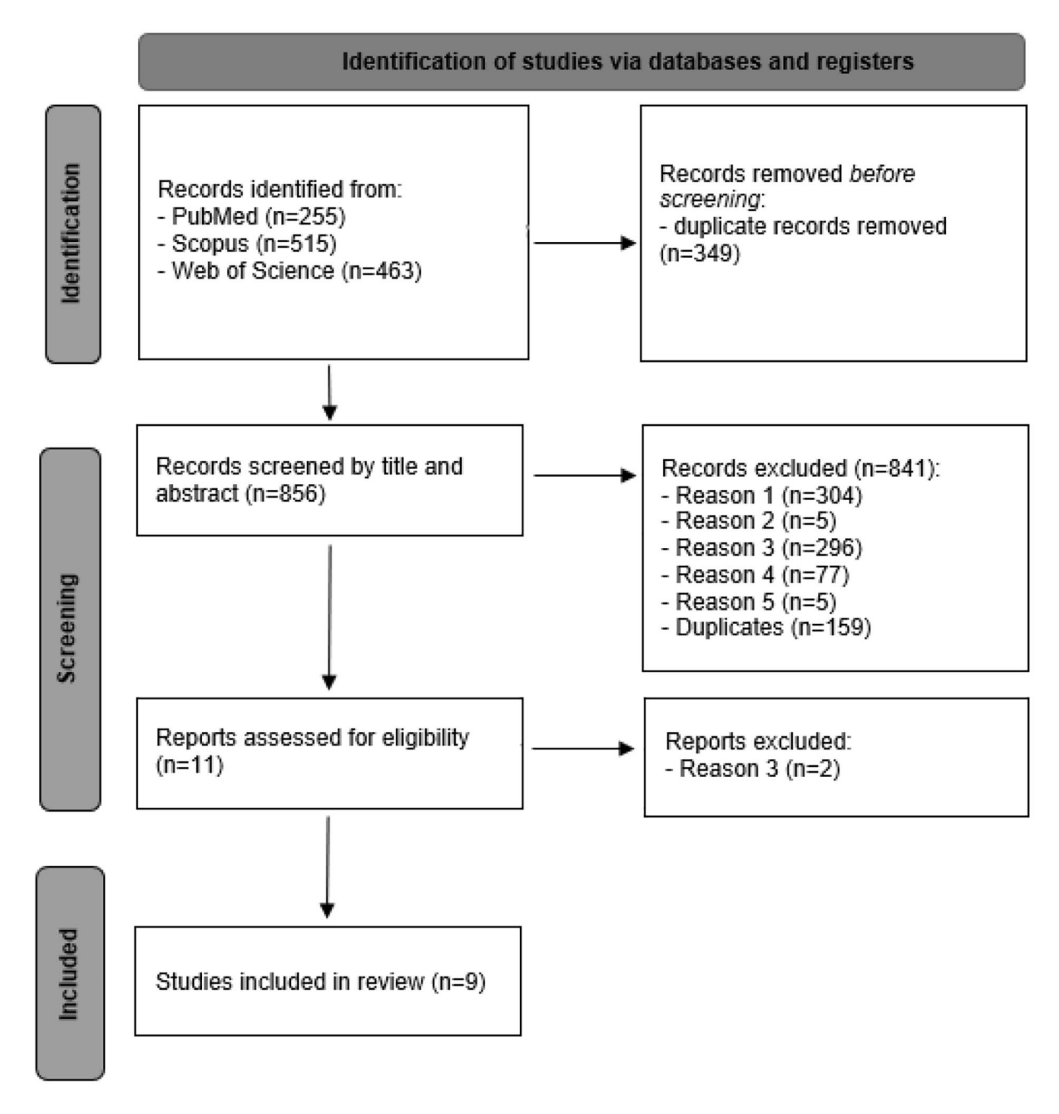
Workflow of the study selection process. Reason 1: Systematic reviews and Meta-analyses, Reviews, Case Reports, Opinion papers or comments, Conference papers, and Letters without results; Reason 2: Not written in English, Portuguese, French, Spanish or Dutch; Reason 3: Not related to oral therapy-induced mucositis; Reason 4: Not related to pre-, pro-, post- or symbiotics; Reason 5: not in-vivo trial simulating oral mucositis

### Data Extraction

Data was independently extracted by two reviewers (LF and IM) using a standardized table. In case of inconsistencies in the data collection process, a third author would resolve it through discussion. The following parameters were retrieved from each primary study: author, year, country, dates of information collection, study type, population characteristics (number of cases, type of treatment, age, control group), study design (such as type of administration and sampling time), biotics characteristics (such as designation and concentration), clinical outcomes, and main conclusions.

### Risk of Bias Assessment

The Cochrane Collaboration tool was used to assess the risk of bias (“RoB”) for randomized controlled trials. The RoB evaluation was conducted separately by two reviewers (LF and IM) and classified as “high risk of bias”, “low risk of bias”, or “unclear risk of bias” if there is any incomplete or unclear data. In case of any inconsistency in the RoB assessment, a third author solved it through discussion (MJA). No RoB assessment was performed on observational before-after studies due to a lack of consensually accepted tools for assessing RoB in those specific studies.

## Results

### Study Characteristics

From a total of 9 studies, 6 were performed on humans [14–19], including 4 randomized controlled clinical trials [15, 17–19], while 3 were performed on animals [20–22]. The countries of origin of the studies were located in Asia [14, 16–20, 22] and Europe [15, 18, 21].

The pre-clinical studies used hamsters and mice which were experimentally irradiated [20, 21] or injected with 5-fluorouracil **(**5-FU) [22]. Regarding the control groups, these studies used a placebo (cryoprotectants and excipients of the formula) [21], saline lavage [20], or drinking water [22]. As for the biotics, only probiotics were tested. One article used a single probiotic *Streptococcus salivarius* K12 [20] and two articles used a combination of probiotics: (i) Caluwaerts et al. [21] used *Lactococcus lactis* sAGX0085 genetically modified to carry erythromycin (Em) and chloramphenicol (Cm) resistance genes and to secrete human Trefoil Factor 1 (htff1) (*Lactococcus lactis* sAGX0085Em + Cm + + htff1); and (ii) Gupta et al. [22] tested *Lactobacillus reuteri* DSM 17,938 and ATCC PTA 5289 strains. Regarding the methods used for administration, a topical application was used in two studies [20, 21] and in one study the probiotic was added to the drinking water [22]. The doses were given in a different posology, as displayed in Table 1. The three articles used macroscopic [20], histologic [20, 22], microbiologic [20], RNA analysis [22], qPCR analysis [22], cell culture [22] and/or immunohistochemistry [21] methods to determine the effect of probiotic use.

**Table 1:** Characteristics and main findings of the included pre-clinical studies

Author, year, country	Study type	Study group					Control group				Measured outcomes	Key results
		Subjects	Sample Size	Condition	Strain and vehicle	Dose, CFU/g	Subjects	Sample Size	Condition	Interventions		
(Caluwaerts, Vandenbroucke et al. 2010), USA and Belgium [21]	Preclinical study	Male Golden Syrian hamsters, 5–6 weeks	15	A single dose of radiation (40 Gy)	AG013 (*Lactococcus lactis* strain sagx05em + Cm+ + htff1), topical application (rinsing fluid)	Single dose 1 × 10^10^ CFU/Dose Day 16 Post-radiation AND Multiple doses 1 × 10^10^ CFU/Dose 3x/day from day 0 to day 16 post radiation	Male Golden Syrian hamsters, 5–6 weeks	18	A single dose of radiation (40 Gy)	AG013 placebo	OM was scored visually by comparison to a validated photographic scale (from 0 to 5).	(1) Study arm had lower severity (shorter period) and course of radiation-induced OM, without systemic exposure. (2) This strain could not survive in systemic circulation, not even under neutropenic conditions.
(Wang, Li et al. 2021), China [20]	Preclinical study	Male BLAB/c mic, 7 weeks old	11	High dose, single fractionated 28 Gy X-ray radiation directly to their head region at a rate of 3.5 12 Gy/min	*Streptococcus salivarius* K12, probiotic solution topical application (with micropipette)	1 × 10^9^ CFU per day	Male BLAB/c mice, 7 weeks old	11	High dose, single fractionated 28 Gy X-ray radiation directly to their head region at a rate of 3.5 Gy/min.	Saline lavage	Macroscopic and histological analyses, 16 S rRNA sequencing. PCR. Anaerobic bacteria cultivation	(1) The *S.salivarius* K12 therapy group experienced less weight loss than the control group. (2) Topical use of *S.salivarius* K12 lowered the intensity of OM considerably. (3) In IR + K12, the relative area of mucositis, including ulcers, was significantly decreased. (4) *S.salivarius* K12 modified the oral microbiome (reduced the amount of *Pasteurella*) and decreased the bacterial load.
(Gupta, Ferreira et al. 2020), Singapore [22]	Preclinical study	Female C3H mice, 10 weeks old	12	5-FU/LR Mice injected with 5-FU and fed with LR in drinking water	*L. reuteri* DSM 17,938 and ATCC PTA 5289 strain (LR added to the drinking water at 1 × 106 Colony forming units (CFU)/ml from day 1)	1 × 10^9^ CFU per day	24 female C3H mice, 10 weeks old	16	5-FU/water Mice injected with 5-FU, and fed with normal drinking water	Normal drinking water	Histopathologic analysis, immunohistochemistry analysis, RNA extraction, qPCR analysis, and cell culture and treatment	(1) Probiotic reduced the severity of chemotherapy-induced OM; (2) *Lactobacillus reuteri* stimulates anti-inflammatory effects and increases resistance to oxidative stress. (3) No systemic bacterial translocation was observed.

OM: Oral mucositis, CT: Chemotherapy, 5-FU: 5-Fluorouracil, RT: Radiotherapy, CFU: colony-forming unit, RIOM: Radiation-induced oral mucositis, BALB/c : an albino, laboratory-bred strain of the house mouse, C3H: LC Strong originated C3H strain in 1920 from a cross of a female Bagg albino × DBA male with selection for high mammary tumor incidence. AG013, mouth rinse formulation of genetically modified *Lactococcus lactis* strain sAGX0085, engineered to secrete human Trefoil Factor 1, cm: chloramphenicol; Em: erythromycin;

Among the 6 human studies included, there were a total of 381 children [14, 16] and adults [15–19] submitted to cancer treatment (207 participants in the intervention group and 174 in the control group), as displayed in Table 2. Participants were submitted to a wide range of oncological treatments that led to the development of OM: chemo-radiotherapy-induced OM was included in two studies [15, 17], chemotherapy-induced OM was reported in two studies [14, 18], chemotherapy combined with hematopoietic stem cell transplantation (HSCT) in one study [16], and in another study, patients were subjected to intensity modulated radiation therapy (IMRT) or concurrent chemo-radiotherapy with cisplatin [19]. These studies used as controls an oral lavage with bicarbonate [15] or a sodium chloride (NaCl) mouthwash [18], a benzidine hydrochloride mouth rinse along with baking soda or distilled water [19], and placebo lozenges (mixture of sugars and salts used as excipients in the active formulation) [17]. In two studies [14, 16], the authors had no control groups and compared the obtained results to other studies [14, 16]. All studies assessed how probiotics affected the severity of OM before and after probiotic intake. No studies assessing pre-, post-, or symbiotics were found. To evaluate the progression of this disease, one article [14] used the Oral Assessment Guide (15), two papers [15, 23] used the Common Terminology Criteria for Adverse Events (CTCAE) 4.0, and the other three [16–18] used the National Cancer Institute’s CTC (NCI CTC) scale. Regarding probiotic administration, in three studies [15–17], *Lactobacillus brevis* CD2 was administered 6 times per day, to be dissolved in the mouth and then swallowed [15–17]. In the other three studies, patients were instructed either to gargle with a mouthwash containing *Lactobacillus casei* and other *Lactobacillus species* (not specified) [14], either to ingest fermented food enriched in probiotics (e.g., kefir) [18], or to ingest 5 ml of an oral suspension containing about 2 million spores of *Bacillus clausii* UBBC − 07, combined with benzidine hydrochloride mouth rinse along with baking soda, twice a day [19].

**Table 2:** Characteristics and main findings of the included clinical studies

Author, year, country	Study type	Study group						Control group			Measured outcomes	Key results
		Age	Sex	Sample size	Type of cancer treatment	Strain and vehicle	Dose, CFU/g	Age	Sample Size	Interventions		
(Christian, Suharsini et al. 2020), Indonesia [14]	Clinical experimental study	Children	-	11	Chemotherapy for leukemia (methotrexate and cytarabine)	Probiotics dissolved in a mouthwash (Probiotics contain mostly *Lactobacillus* species and *Lactobacillus casei*). Patients had to gargle for 1 min with the probiotic twice/day for 7 days. Patients continue gargling for 14 days.	-	Compared results to other studies	Compared results to other studies	Compared results to other studies	OAG	(1) There was a decrease in the OAG score 7 and 14 days after gargling with probiotics.
(De Sanctis, Belgioia et al. 2019), Italy [15]	Multicentric, phase III, open-label, randomized controlled trial	Mean age: 58,4 (34–74)	26 males and 6 females	32	IMRT + Cisplatin-based chemotherapy + cetuximab. Neo-adjuvant chemotherapy (docetaxel, cisplatin, and 5-FU) every 21 days for patients with nasopharyngeal cancer.	*Lactobacillus brevis* CD2 lozenges are to be dissolved in the mouth and then swallowed.	2 × 10^9^ CFU/ml, 6 times/day, every 2-3 h, from the first day of RT to 1 week after the end of treatment	Mean age: 60 years	36	Bicarbonate mouthwash, 3x per day	CTCAE 4,0 for OM and QOL questionnaires	(1) There was no difference in the incidence of severe OM between intervention and control groups during RT. (2) No benefit of the use of *L. brevis* CD2, compared to the control arm, in reducing the incidence of severe OM, significant improvement of QOL, or acute toxicities such as weight loss, pain, and dysphagia.
(Sharma, Rath et al. 2012), India [17]	A randomized, double-blind, single-center, placebo-controlled study	Mean age: 52,35+/-9,43	94 males and 7 females	93	HNSCC - radical radiotherapy at a dose of 70 Gy in 35 fractions over 7 weeks by linear accelerator + chemotherapy of cisplatin 40 mg/m^2 weekly for 7 doses	*Lactobacillus brevis* CD2 lozenges are to be dissolved in the mouth and then swallowed.	2 × 10^9^CFU/ml, 6 times/day, every 2-3 h, from the first day of CRT to 1 week after the end of treatment	Mean age: 50,09+/- 10,04	95	Placebo lozenges: a mixture of sugars and salts used as excipients in the active formulation	NCI CTC version 2.0 + QOL assessed using the FACT-HN questionnaire, version4	(1) No statistically significant improvement in QOL in the *L. brevis* CD2 arm compared to the placebo. (2) No significant adverse events attributed to the study product or placebo were identified. (3) The proportion of patients with grade III or IV mucositis was lower in the *L. brevis* arm (52%) than in the placebo arm (77%). (4) The proportions of patients with grade I and II mucositis were similar (19% versus 15%). (5) Study arm had considerably fewer individuals needing analgesics for mucositis-related discomfort.
(Sharma, Tilak et al. 2016), India [16]	Single-arm, single-center, phase II clinical study	Mean age: 29 years (10–70)	19 males and 12 females	31	High dose of chemotherapy + HSCT	Lactobacillus brevis CD2 lozenges to be dissolved in the mouth.	> 2 × 10^9^ viable cells of L. brevis CD2, four to six lozenges per day (mean: 3), one lozenge to be taken every 2 to 3 h, beginning from 4 to 7 days before initiation of chemotherapy and continuing until resolution of OM until the 24th day after the end of treatment	Compared results to other studies	Compared results to other studies	Compared results to other studies	NCI-CTCAE scale.	(1) Only 19.4% of patients experienced severe OM (grades 3 and 4) (2) Median period for mucositis development and resolution was 6 days and 8 days, respectively. (3) *L. brevis* appears to be both safe and effective in avoiding OM.
(Topuz, Derin et al. 2008), Turkey [18]	Randomized, prospective, observational study	Mean age: 51 years (19–75)	12 males and 5 females	17	5-FU-based CT protocol	Oral lavage with kefir, and ingestion	Oral lavage (250 ml) with kefir to be swallowed after gargling. To be repeated on the first 5 days of each CT cycle, twice a day after meals.	58 (34–72)	20	Oral lavage with 0.09% NaCl twice a day on the first 5 days of each CT cycle	NCI- CTCAE scale and Oral Assessment Guide.	(1) Kefir had no significant influence on serum levels of inflammatory cytokines and no anti-mucositis role. (2) Kefir only inhibited *Staphylococcus epidermidis*.
(Mirza et al.; 2022), India [19]	Randomized, double-blind, placebo-controlled, parallel study	51 (19–75)	22 males and 1 female	23	Thirty-eight patients underwent definitive surgical resection before adjuvant RT. Eight patients received IMRT with concurrent chemotherapy with weekly cisplatin (40 mg/m^2^)	Oral suspension of *Bacillus clausii* UBBC07 containing 2 billion spores	5 ml (2 billion spores) 2x/ day, for 30 days or until completion of total fractions of radiation	31–60	23	Standard treatment (Benzidine hydrochloride mouth rinse along with baking soda) and 5 ml of distilled water twice daily	OM was assessed using the CTCAE scale. (1–4)	(1) Arm study had a shorter duration of remission and a smaller proportion of patients with high-grade OM (grade II and up). (2) Arm study had no RT-related side effects, such as diarrhea. (3) Findings suggest that radiotherapy may be complemented with probiotics to alleviate RIOM symptoms.

OM: Oral mucositis, CT: Chemotherapy, 5-FU: 5-Fluorouracil; RT: Radiotherapy, IMRT: Intensity modulated radiation therapy; HSCT: Hematopoietic stem cell transplantation; OAG: Oral Assessment Guide, CFU: colony-forming unit, CTCAE: Common Terminology Criteria for Adverse Events, NCI-CTCAE: National Cancer Institute Common Terminology Criteria for Adverse Events, RIOM: Radiation-induced oral mucositis, QOL: Quality of Live

### Risk of Bias Within Studies

The risk of bias (RoB) was assessed to the four clinical trials retrieved, as displayed in Table 3. One randomized clinical trial (RCT) was marked as having a low RoB [17], while other two were marked as unclear [18, 19] and one having a high RoB [15]. The study of Christian et al. [14], although being designed as a clinical trial, did not have a control group, and cannot be considered an RCT. In the other studies, two uncontrolled before-after studies with a low number of participants [14, 16] and three studies in animals [20–22], the assessment of RoB was not feasible.

**Table 3:** Risk of bias

	1.1 Random sequence generation	1.2 Allocation concealment	2.1 Selective reporting	3.1 Other sources of bias	4.1 Blinding (participants and personnel)	5.1 Blinding (outcome assessment)	6.1 Incomplete outcome data
De Sanctis, Belgioia et al. 2019 [15]	?	+	?	?	-	-	-
Sharma, Rath et al. 2012 [17]	+	+	+	+	+	+	+
Topuz, Derin et al. 2008 [18]	?	?	?	?	?	?	+
Mirza et al. 2022 [19]	+	+	+	?	?	?	+

**+ -** low risk of bias, **-** - high risk of bias,**?** - unclear risk of bias

## Results of Individual Studies

All the included pre-clinical studies [20–22] described probiotic interventions as effective in reducing OM severity. Four included studies in humans [14, 16, 17, 19] described that probiotic intervention was effective in reducing and preventing the degree and severity of oral mucositis in patients undergoing cancer therapy either radiotherapy, hematopoietic stem cell transplantation, or chemotherapy. Meanwhile, two studies reported that the difference in the incidence of oral mucositis between the intervention and control groups was not significant [15, 18].

Concerning the pre-clinical studies, Caluwaerts et al. [21] found that a mouth rinse containing 10^8^ or 10^10^ CFU/dose of *Lactococcus lactis* sAGX0085Em + Cm + + htff1 (coded AG013) significantly reduced the period of severe OM in hamsters. It is noteworthy that AG013 was qualified as more effective than a mouth rinse or an oral spray containing high amounts of the therapeutic peptide itself because of the longer-lasting contact with the mucosa. In addition, the authors found that in single- and multiple-dose pharmacokinetic (PK) studies in healthy and irradiated hamsters, living and metabolic active AG-X0085Em + Cm + bacteria could be recovered from the oral cavity up to 24 h post-dosing, but there was no exposure beyond the mucosal compartment. These findings supported that the administration of AG013 to OM patients at risk of developing neutropenia is safe.

Wang et al. [20] stated that topical application of *S. salivarius* K12 significantly reduced the severity of OM in mice, finding that the relative area of mucositis including ulcers was significantly reduced in the intervention group (p < 0.001) and described the capacity of *S. salivarius* K12 in modulating the oral microbiome through the inhibition of oral anaerobes (reduced *Pasteurella*, *Corynebacterium*, *Porphyromonas*, and *Staphylococcus*). Moreover, in this study it was observed that in the group of irradiated (IR) mice treated with *S. salivarius* K12, the relative area of mucositis (including ulcers) was lower (9.03%) compared to the IR mice treated with a saline solution (77.42%), and had restored the integrity of the lingual mucosa, showing a thicker mucosa and basal layer epithelial cellularity. Finally, the weight of mice who received irradiation decreased sharply (-12,05 g), while *S. salivarius* K12 treatment lessened the body weight loss (-8.33 g).

Gupta et al. [22] stated that the tested *L. reuteri* strains (LR) were effective in reducing OM severity, *as* it was found that the epithelial damage was less severe in the group injected with 5-FU and fed with LR in drinking water (5-FU/LR group) (p < 0.001) and had higher expression of Ki-67 protein (proliferation marker) in basal epithelial cells (p < 0.001) resulting in a higher epithelial regeneration, comparing to the 5-FU/water group. Additionally, it was shown that *L. reuteri* reduced oxidative stress through the nuclear factor E2-related factor-2 (Nrf-2) signaling. Concerning the safety of these strains, the probiotic administration did not result in systemic bacterial translocation, suggesting that these *L. reuteri* strains are safe for administration during chemotherapy.

Regarding the included clinical studies, Sharma, Tilak et al. [16] reported that the use of *L. brevis* was safe and effective in preventing OM in patients undergoing high-dose chemotherapy and autologous stem cell transplant (HSCT). *L. brevis* was administered to all participants as there was no control group. Fluconazole and itraconazole prophylaxis were given to all patients, and acyclovir prophylaxis to transplant patients. The results showed that around 19.4% of the patients developed severe OM, 58.1% of the patients presented mild to moderate OM, and a total of 22.5% of patients did not develop OM. The time to onset OM was 6 days and for resolution/healing, it took 8 days after the day of stem cell infusion.

Sharma, Rath et al. [17] stated that administration of *Lactobacillus brevis* CD2 lozenges in head and neck squamous cell carcinoma (HNSCC) patients enduring radiotherapy and concurrent cisplatin-based chemotherapy, reduced the incidence of severe OM (52% incidence in the intervention group versus 77% in the placebo group). It was also observed that the administration of this probiotic was able to reduce the occurrence of OM, as there were more remaining free OM patients in the intervention arm (28% vs. 7%). Regarding OM severity, 28% of the patients in the study arm did not develop OM, 19% developed mild to moderate mucositis, and 52% developed severe OM. On the other hand, 7% of the patients in the placebo arm did not develop OM, 15% developed mild to moderate mucositis (p < 0.001), and 77% developed severe OM (p < 0.001). The median time to the onset of mucositis was higher in the intervention group (22 days) than in the control group (18 days). However, the median time to heal mucositis was 43 days in both groups. It was also mentioned that no serious adverse events were observed when using *L. brevis* CD2 probiotic. Additionally, a higher percentage of patients in the *L. brevis* CD2 group (p = 0.001) completed the planned treatment (92% vs. 77%), not showing evidence of grade II nausea and vomiting and no non-compliance to the cancer treatment. Although there was a trend towards improvement in QOL in the *L. brevis* CD2 arm compared to the placebo, it was not statistically significant. However, it was also observed that, compared to the placebo group, with the use of *L. brevis* CD2, fewer patients required analgesics for mucositis-associated pain (p = 0.02). Moreover, among patients who were able to complete the anticancer treatment, the requirement for parenteral nutrition or Ryle’s tube insertion trended lower in the *L. brevis* CD2 arm.

Conversely, De Sanctis et al. [15] found that *L. brevis* CD2 had no effect in reducing the incidence of severe OM induced by intensity-modulated radiation therapy (IMRT) and concomitant cisplatin-based chemotherapy. De Sanctis et al. [15] reported that 40.6% of the patients in the intervention group and 41.6% of the patients with sodium bicarbonate mouthwash (control group) developed severe OM. It was also noticed that there was a statistically significant tendency for weight loss during concurrent therapy compared to baseline (p < 0.01), independently of the intervention or control arm. It was also noted that dysphagia was greater in the intervention arm (p < 0.05) and there was no difference between groups regarding pain evolution. Although probiotics were considered ineffective in reducing or preventing OM, it was stated that there was no serious adverse event related to *L. brevis* CD2 lozenge administration.

Christian et al. [14] concluded that there was a statistical difference (p < 0.05) in Oral Assessment Guide (OAG) before and after gargling with probiotics containing *Lactobacillus* species (not specified) and *Lactobacillus casei* in children with leukemia submitted to chemotherapy. It is noteworthy that there was a statistically significant decrease in OAG score between days 7 and 14 after gargling with the probiotics. Therefore, they concluded that probiotics could be an alternative therapy and prevention for oral mucositis.

According to Mirza et al. [19], taking *Bacillus clausii* UBBC − 07 twice a day allowed a substantial increase in the median time of mucositis onset (10 days in the intervention group versus 8 days in the control group; p < 0.01) and a significant decrease in the median time for remission (12 days in test and 14 days in control groups; p < 0.05). Additionally, it was described that, in the intervention group, 8 out of 23 patients had a significantly lower incidence of higher-grade OM (grade III or higher) compared to the control group (16 out of 23 patients; p < 0.05). In contrast to the placebo group, the test group did not experience diarrhea as a side effect of RT. Additionally, no adverse events associated with *Bacillus clausii* were observed.

Lastly, Topuz et al. [18] reported that kefir use was considered ineffective in decreasing OM severity. In fact, during chemotherapy, mucositis incidence increased significantly with increasing chemotherapy cycles in the kefir group (p = 0.009). However, this was not the case for the control group receiving an oral lavage with 0.09% NaCl (p = 0.29). When the two were compared for incidence of OM during therapy, no statistical significance was detected, as 72.7% of the intervention group did not develop OM (versus 78.3% of the patients in the control group). Additionally, the authors found that during chemotherapy, serum proinflammatory cytokines did not change significantly.

## Discussion

Despite advances in medical therapy, the current knowledge in the area of prevention and treatment of therapy-induced oral mucositis is very limited. As previously stated, the management of oral mucositis remains mostly symptomatic and there is no effective strategy for its prevention or treatment [24]. Thus, there is a need to find new alternatives or complementary therapies. Consequently, knowing the positive effect of biotics in other diseases, and that some bacteria strains can modulate the epithelial cells, barrier function, mucosal immunity, and macrophage signaling pathways influencing cytokine production, we may consider biotics as a therapeutic possibility [24, 25]. As the occurrence of oral mucositis seems likely to happen after cancer therapy, the primary concern is to prevent its onset and progression. Probiotics have successfully been used to prevent and reduce mucositis severity in clinical and preclinical studies, but no studies were found using pre-, post- and symbiotics. *Lactobacillus* species intake, specifically *L. brevis* CD2 [16, 17], *L. casei* [14], and *L*. *reuteri* [22], as well as *Streptococcus salivarius* K12 [20], *Bacillus clausiI* UBBC − 07 spores [19] and *Lactococcus lactis* (AG013) [21] appear to be associated with a decrease in OM incidence and severity.

There are several mechanisms described to explain the effectiveness of these probiotic strains. As for *Lactobacillus* spp., these strains presented promising results and seemed to activate important anti-inflammatory mechanisms, which would benefit OM patients. For instance, Sharma, Rath et al. [17] explained that *L. brevis* CD2 produces high levels of arginine deiminase and sphingomyelinase which compete with nitric oxide synthase, leading to a reduction in the levels of some of the inflammatory factors (cytokines interleukin (IL)-1a, IL-6, IL-8, tumor necrosis factor-alpha (TNF-α), interferon-gamma (IFNγ), prostaglandin E2 (PGE2) and matrix metalloproteinases). Furthermore, bacterial sphingomyelinase can hydrolyze the platelet-activating factor (PAF), a potent inflammatory cytokine, and is known to be associated with oral mucositis in radiation therapy [17]. Lee et al. [26] reported that *Lactobacillus casei* significantly decreased TNF-α, and IL-6, and adhered to surface molecules by suppressing the signaling pathway of IL-6 and TNF-α. Amdekar et al. [27] mentioned that *L. casei* induces ciclo-oxigenase-2 (COX-2) inhibition, having an antiarthritic effect. Lastly, Gupta et al. [22] reported that *Lactobacillus reuteri* DSM 17,938 and ATCC PTA 5289 strains seem to be capable of modulating the host inflammatory response by reducing pro-inflammatory cytokine response (e.g., TNF-α, IL-beta, and Myeloperoxidase (MPO)) and of increasing key antioxidant genes (i.e., superoxide dismutase-1 (SOD-1), glutathione peroxidase-1 (GPx-1), and heme oxygenase-1 (HO-1)). In recent literature, *Lactobacillus reuteri* has been associated with reduced gingival inflammation and a decrease in pathogens associated with periodontitis [28]. Mu et al. [29] demonstrated that *L. reuteri* can produce antimicrobial molecules, such as organic acids, ethanol, and reutein, inhibiting the colonization of pathogenic microbes and remodeling commensal microbiota. The immunomodulatory effects of the probiotic *Bacillus clausii* in intestinal health are well established, as well as its ability to treat gastrointestinal discomfort. However, there may be further advantages in other therapeutic fields that are only now being identified [30]. For instance, according to Nirmala et al. [31], using *B. clausii* as a local adjuvant greatly decreases the symptoms of oral candidiasis and recurrent aphthous ulcers. Wang et al. [20] mentioned that *S. salivarius* K12 can modulate the oral microbiome, reducing the abundance of anaerobic bacteria and ulceration, increasing the thickness of the tongue mucosa and the density of basal cells, and enhancing basal cell proliferation and attenuating apoptosis. Burton et al. [32] showed in vitro that *S. salivarius* K12 suppressed the growth of different strains of bacteria implicated in halitosis, enhancing the capacity to modulate the microbial ecosystem. Caluwerts et al. [21], genetically modified *Lactococcus lactis* strain sAGX0085 engineered to secrete human Trefoil Factor 1. The authors stated that TFF1 was found as a gastric tumor suppressor and, at the cellular level, TFF1 promotes cell differentiation while limiting cell proliferation and apoptosis [21]. Strains of *Lactococcus lactis* have only recently been explored for their possible cytotoxic effects against human cancer cell lines [33] and anti-inflammatory properties and capacity in preventing 5-FU-induced gut dysbiosis [34]. In summary, the anti-inflammatory, immunomodulatory, and antioxidant properties of probiotic strains would be of great value considering the five-step pathogenesis model of OM proposed by Sonis [3], as they may protect against the negative effects of radiotherapy and chemotherapy on the oral mucosa.

When evaluating the effectiveness of a probiotic, it is essential to understand whether there is a trend towards the improvement of the QOL. The WHO defines the QOL as an individual’s perception of their position in life in the context of the culture and value systems in which they live and in relation to their goals, expectations, standards, and concerns. Regarding the QOL, the results between pre-clinical and clinical studies differed. In other diseases, some studies reported an improvement in the QOL of individuals who were given probiotics. This is the case of Waal et al. [33], in which the authors found an improvement in QOL in 66% of the patients suffering from ulcerative colitis taking a probiotic. Nevertheless, more studies are needed to evaluate the impact of probiotic intake in the QOL of OM patients.

Considering that all cancer patients are considered immune-depressed and some HNC patients develop neutropenia, the evaluation of the safety of probiotic strains is essential. Although most of the above-mentioned studies reported that probiotics are safe, the Agency for Healthcare Research and Quality (AHRQ) [35], in 2011, concluded that there is still a lack of evidence to confidently recommend probiotic interventions to the healthcare and nutrition communities. According to the World Health Organization in 2002, probiotics may theoretically be responsible for systemic infections, deleterious metabolic activities, excessive immune stimulation in susceptible individuals, and gene transfer [36]. Nevertheless, the AHRQ [35] also affirmed that the lack of adverse events supports the safety of probiotics. The European Food Safety Authority (EFSA) has already recognized the health benefits that are pertinent to the effects of probiotics in some health conditions in 2021, under the Nutrition & Health Claims Regulation [37]. However, EFSA has not yet published guidelines concerning the safety of probiotic use, specifically in immune-depressed patients [37]. As such, further studies are necessary to evaluate the safety concerns of probiotic treatment in immunocompromised patients.

Although our findings support the conclusion that probiotics may reduce the onset and severity of cancer therapy-induced OM, some potential limitations should be addressed. Firstly, the number of studies examined was small (n = 9) and with heterogeneous study designs. For example, the difference in findings between the studies from De Sanctis et al. [15], Sharma, Tilak et al. [16], and Sharma et al. [17], where authors tested *L. brevis* strains but only the two studies of Sharma et al. [16, 17] showed positive effects on oral mucositis. These outcomes could be explained by different cancer treatments, and different control groups (sodium bicarbonate mouthwash versus placebo lozenges, respectively) which could induce a lower rate of severe OM. Referring to the cancer treatment, it is important to state that IMRT may have improved tolerance to concomitant chemoradiotherapy (RCHT) or intraoperative radiotherapy (bioRT), reducing the effectiveness of *L. brevis* CD2. Secondly, cancer type and treatment differed across studies. For instance, Sharma, Rath et al. [17] included patients receiving radical radiotherapy at a dose of 70 Gy and chemotherapy of cisplatin, while Sharma, Tilak et al. [16] included patients in a chemotherapy regime with hematopoietic stem cell transplantation (HSCT). Third, the probiotic composition, posology, mode of administration, and additional treatments varied across studies. For example, Topuz et al. [18] considered the use of kefir ineffective in decreasing OM severity. However, kefir had a short permanence in the oral cavity since it was ingested, while in other studies the probiotics were dissolved in the mouth before ingesting [15–17] or applied as a mouthwash for a defined period [14] or applied as an oral suspension [19], resulting in a long-lasting direct contact with the oral cavity. Thus, the mode of administration and the time of contact could influence the impact of the probiotic on the progression of OM. It should also be noted that some studies used sodium chloride or sodium bicarbonate in the control group [19]. Both sodium bicarbonate and sodium chloride are known to be effective in treating and reducing the severity of oral mucositis [38] and promoting healthy gum and improving oral ulcer healing [39], respectively. The use of these components could influence the results due to their influence on oral physiology. It should also be noted that the quality of the studies is overall low because there are unclear aspects and only one study [17] was considered to have a low risk of bias. To summarize, probiotics appear to be a safe treatment option for cancer therapy-induced OM, but additional research is needed to assure their efficacy and security as well as to better define the most efficient posology and formulation. Moreover, would be also relevant to explore other biotics formulations, such as pre-, post-, and symbiotics.

## Conclusions

In conclusion, the findings of this systematic review suggest that probiotic supplementation could potentially reduce the incidence of therapy-induced oral mucositis or alleviate its symptoms in chemotherapy or radiotherapy patients. The available evidence, however, is limited and marred by significant heterogeneity across studies. Taking these findings into account, we suggest further research particularly regarding the probiotic strains of *L. brevis* CD2, *L*. *reuteri*, *L. casei*, S. *salivarius* K12, *B. clausii* UBBC − 07 spores, and *Lactococcus lactis* (AG013), as these presented promising results. Further recommendations for future studies include the use of probiotic combinations, bearing in mind possible beneficial interactions, as well as standardized control groups. It is also critical to determine the proper probiotic posology and formulation for better results and safety and to develop guidelines for safe probiotic use, particularly in immunocompromised patients.

## Data Availability

Not applicable.

## Declarations

### Conflit of Interest

None declared.
